# Iridoid Glycosides Fraction of *Folium syringae* Leaves Modulates NF-*κ*B Signal Pathway and Intestinal Epithelial Cells Apoptosis in Experimental Colitis

**DOI:** 10.1371/journal.pone.0024740

**Published:** 2011-09-13

**Authors:** Xin Liu, Jian Ming Wang

**Affiliations:** 1 College of Pharmaceutical Sciences,Zhejiang University, Hangzhou, China; 2 Academy of Traditional Chinese Medicine, Heilongjiang University of Chinese Medicine, Harbin, China; Charité-University Medicine Berlin, Germany

## Abstract

**Background and Aims:**

Iridoid glycosides (IG), the major active fraction of *F. syringae* leaves has been demonstrated to have strong anti-inflammatory properties to ulcerative colitis (UC) in our previous study. The aim of this study was to investigate whether IG modulates the inflammatory response in experimental colitis at the level of NF-*κ*B signal pathway and epithelial cell apoptosis.

**Methods:**

UC in rats was induced by administration with dextran sulfate sodium (DSS) in drinking water. The inflammatory damage was assessed by disease activity index (DAI), macroscopic findings, histology and myeloperoxidase (MPO) activity. The effect of IG on pro-inflammatory cytokines TNF-*α*, IL-8, COX-2 and regulatory peptide TGF-β1 was measured. Epithelial cell apoptosis and the protein and mRNA expressions of Fas/FasL, Bcl-2/Bax, caspase-3, NF-*κ*B p65, I*κ*B*α*, p-I*κ*B*α* and IKKβ were detected by TUNEL method, immunohistochemistry, Western blotting and real-time quantitative PCR, respectively.

**Results:**

IG significantly ameliorated macroscopic damage and histological changes, reduced the activity of MPO, and strongly inhibited epithelial cell apoptosis. Moreover, IG markedly depressed TNF-α, IL-8, COX-2 and TGF-β1 levels in the colon tissues in a dose-dependent manner. Furthermore, IG significantly blocked of NF-*κ*B signaling by inhibiting I*κ*Bα phosphorylation/degradation and IKKβ activity, down-regulated the protein and mRNA expressions of Fas/FasL, Bax and caspase-3, and activated Bcl-2 in intestinal epithelial cells.

**Conclusions:**

These results demonstrated for the first time that IG possessed marked protective effects on experimental colitis through inhibition of epithelial cell apoptosis and blockade of NF-*κ*B signal pathway.

## Introduction

Ulcerative colitis (UC), the major forms of inflammatory bowel disease (IBD), is an immunologically mediated chronic intestinal disorder. The disease commonly follows a chronic relapsing course with clinically quiescent periods followed by bouts of severe intestinal inflammation, which are characterized by abdominal pain, diarrhea, rectal bleeding and weight loss [1]. Furthermore, prolonged and chronic UC may progress to colorectal cancer [2]. Despite substantial progress has been made in the treatment of UC along the immune and inflammatory pathways, no definitive therapies with a nonrelapsing cure rate are available for this disorder until now, and limiting drug-induced toxicity is a continuous challenge [3], [4]. Traditional therapeutic modalities are used for UC including anti-inflammatory therapy (5-aminosalicylic acid and corticosteroids), some immunomodulators (azathioprine, 6-mercaptopurine and cyclosporine) [5]. However, these drugs have demonstrated unsatisfactory results and the recrudescence rates of IBD are rather high. Therefore, it is challenging to develop new and specific therapies for the treatment of IBD.

Although the exact etiology and pathogenesis of IBD are not fully defined up to now, a growing body of work suggests that inflammatory response and intestinal epithelial cell (IEC) apoptosis mainly participate in the pathogenesis of IBD [1], [6]. The abnormal mucosal immune and inflammation responses in IBD are predominantly characterized by increased synthesis of pro-inflammatory cytokines, the activation of neutrophils, and enhanced formation of reactive oxygen and nitrogen species. These mediators can activate the nuclear factor kappa B (NF-κB) pathway, modulating a number of different steps in the inflammatory cascade [7], [8]. These include production of pro-inflammatory cytokines such as tumour necrosis factor alpha (TNF-α), interleukin-1β (IL-1β), interferon-γ (INF-γ), IL-6, IL-8, and IL-12 in different cell-types, degranulation of neutrophils, as well as the expression of important inflammatory proteins such as cyclooxygenase-2 (COX-2) and inducible nitric oxide synthase (iNOS) [9]–[11]. Therefore, imbalance between pro-inflammatory and anti-inflammatory cytokines and inflammatory proteins expression, plays an important role in the modulation of intestinal immune system and contributes to the inflammatory cascade in the pathological process of colitis [12]. On the other hand, the pathogenesis of UC involved in the abnormality of apoptosis. Growing evidence indicated that the nuber of apoptotic epithelial cells is increased during active UC, which may lead to an alteration of the epithelial barrier function resulting in pathogenic microorganism infiltration [6], [13]. It has been reported that different mechanisms are involved in the induction of apoptosis. One pathway is mediated by the interaction between cell surface death receptor Fas and its specific ligand FasL. Such an interaction leads to the formation of the death-inducing signalling complex and activation of caspase-8 and caspase-10, two initiator caspases that in turn activate downstream effector caspase-3 [14]. Another is mediated by proapoptotic signals at the mitochondria level including members of Bcl-2 family and caspase-9 [15]. Transforming growth factor-β1 (TGF-β1) counteracts TNF-α and regulates cFLIP protein acting as a negative regulator of caspase-8 and thereby inhibits Fas-mediated apoptosis in mucosal inflammation that is essential for wound healing and tissue repair [16], [17]. Importantly, TGF-β is known to be one of the most potent cytokines in the regulation of mediators (TGF-α, EGF, IL-1, IL-2, IFN-γ) and plays an important role in the prevention of intestinal epithelial destruction [18], [19]. TGF-β1 expression is increased parallel to the increase of pro-inflammatory cytokine secretion in patients with UC and Crohn's disease (CD) [20]. NF-*κ*B mediates the IL-1β induction of TGF-β1 gene expression and NF-*κ*B RelA antisense oligonucleotides suppress TGF-β1 mRNA expression [21]. There are also studies implying that successful treatment of UC-related mucosal injury results in decrease of TGF-β1 level in plasma and intestinal mucosa [22]. In this regard, the regulation of inflammatory response and IEC apoptosis may be a promising therapeutic modality for UC.

NF-*κ*B signal pathway plays a pivotal role in regulating the production of pro-inflammatory cytokines and apoptosis of IEC in UC, which contribute to cytokine-mediated mucosal tissue damage, leading to a breakdown in the mucosal barrier [23], [24]. Increased NF-κB activation has been detected in the mucosa of patients with IBD and in a murine colitis model, and inhibition of NF-*κ*B with a specific p65 antisense oligonucleotide is effective in preventing experimental models of IBD and efficiently down-regulates cytokine production by intestinal macrophages from Crohn's disease (CD) patients [25], [26]. NF-*κ*B can be activated by diverse stimuli (e.g., pro-inflammatory cytokines, microbes and microbial products, and oxidative stress) that signal its activation through the catalytic I*κ*B kinase β (IKKβ) [23], [27]. IKKβ phosphorylates NF-*κ*B-bound I*κ*Bs in the cytoplasm and targets their degradation, thereby leading to subsequent release of NF-*κ*B dimmers, which then translocate from the cytoplasm to the nucleus and activate the transcription of multiple *κ*B-dependent target genes [28], including pro-inflammatory cytokines (TNF-*α*, IL-1*α*, IL-6, IL-8, IL-12, MCP-1, interferon-γ), death and survival proteins (Bcl-2, Bcl-xl, Bcl-xs, Bax, p53, Myc, Fas), intercellular adhesion molecules (ICAM), COX-2 and iNOS [29], [30]. Growing evidence reveals that the inhibition of NF-*κ*B activity by either directed blockade of RelA (p65) or suppression of I*κ*Bα degradation or IKKβ activity may lead to alleviating the severity of intestinal inflammation [26], [31]. Therefore, the modulation of NF-κB signaling pathway could be the main target for the treatment of IBD.

*Folium syringae* leaves have been used in herbal medicines to treat inflammatory intestinal disease such as acute enteritis and bacillary dysentery in China for a long time. Iridoid glycosides (IG) is the main active fraction extracted from *F. syringae* leaves, with high content of syringopicroside [32]. Although our previous studies indicate that IG exert conspicuous anti-inflammatory effects on UC *in vivo* by scavenging reactive oxygen species (ROS) and inhibiting relative pro-inflammatory cytokines [33]. The molecular mechanisms of IG involved in protection against UC are still not entirely clear. In the present study, we further explored whether its mechanism was associated with modulating NF-*κ*B signaling pathway and IEC apoptosis. The protective effects of IG on dextran sulfate sodium (DSS)-induced colitis were assessed by macroscopic score and histological analysis as well as by determination of inflammation markers such as MPO activity and the mRNA expressions of pro-inflammatory cytokines such as TNF-α and IL-8. The activity of COX-2 and TGF-β1 were also evaluated. In order to elucidate the probable mechanisms of IG in ameliorating inflammatory injury in experimental colitis, the anti-inflammatory effects of IG on activation change of NF-*κ*B signaling pathway and the relative expressions of serial genes involved in IEC apoptosis were evaluated by immunohistochemistry, western blotting and real-time quantitative PCR, respectively.

## Materials and Methods

### Animals and reagents

 Male Sprague-Dawley rats weighing 200–220 g, were supplied by Slaccas Laboratory Animal Co. Ltd. (Shanghai, China). The rats were maintained in standard cages in a controlled room (temperature 24–25°C, humidity 70–75%, lighting regiment of 12L/12D) and fed with standard rodent diet. All animal experiments were approved under animal protocol number SCXK (Zhe) 2008-0033 by Zhejiang Medical Laboratory Animal Administration Committee. Experimental animals were treated according to Guideline of Laboratory Animal Care from Chinese Ministry of Science and Technology in 2006 (Available from: http://www.most.gov.cn/fggw/zfwj/zfwj2006/200609/t20060930_54389.htm).

*F. syringae* leaves were collected in Heilongjiang Province, China, in September 2008. Voucher specimens (No. 20080916) of this material was identified by Professor Jianming Wang, and deposited at Heilongjiang University of Chinese Medicine. The iridoid glycosides fraction was purified using D-141 macroporous adsorption resin column from *F. syringae* leaves based on previously described procedures [32]. The content of syringopicroside in the iridoid glycosides fraction reached 55.74%.

No specific permits were required for the described field studies. The field studies did not involve endangered or protected species.

Salicylazosulfapyridine (SASP) was purchased from Sine Pharmaceutical Co. Ltd. (Shanghai, China). DSS was provided by MP Biomedicals (M.W. = 36–50 kDa, USA). Apoptosis detection kit was supplied by Boster Bio-engineering limited company (Wuhan, China). Bax kit was purchased from Santa Cruz Biotechnologies (USA). The primers for real-time quantitative RT-PCR were synthesized from GeneCore Biotechnologies Co. Ltd. (Shanghai, China).

### Induction of colitis and experimental design

Rats (n = 60) were adapted for 1 week and randomly assigned to 6 groups (n = 10). Drinking water containing 4% dextran sulfate sodium (DSS) was provided for 1 week to induce colitis [34]. Following 7 days of DSS administration, IG (80, 160 and 240 mg/kg) were suspended in 0.9% saline solution and administered twice daily by oral gavage for 14 days, respectively. Positive control group received SASP in a dose of 150 mg/kg twice daily. Normal and model groups received the vehicle in a comparable volume (10 ml/kg body weight), respectively.

### Macroscopic assessment and histological study of colon damage

The rats were checked daily for body weight, behavior, stool consistency and the presence of gross blood in stool. At the end of the experimental period, rats were sacrificed using an overdose of anesthetic. The entire colon was excised from the cecum to the anus and opened longitudinally. Colon length as an indirect marker of inflammation was measured. Macroscopic damage was assessed using a validated scoring system with slight modifications [35], [36]. The numerical rating score were as follows: 0, no inflammation; 1, local hyperemia without ulcers, and/or stool consistency; 2, ulceration without hyperemia; 3, ulceration and adhesions at one site; 4, two or more sites of inflammation and ulceration extending >1 cm; and 5, ulceration extending more than 2 cm.

Colonic tissues were fixed in 4% buffered paraformaldehyde, then dehydrated through graded concentrations of ethanol, subsequently embedded in paraffin, and finally sectioned in 4 µm thick sections. After dewaxing and rehydration, the sections were stained with hematoxylin-eosin (H&E) according to standard procedures for histological evaluation. The colonic pieces were collected from inflamed portions after samples were taken for histological examination and frozen in liquid nitrogen to quantify biochemical parameters.

### Determination of myeloperoxidase (MPO) activity

Myeloperoxidase (MPO) activity was determined with the O-dianisidine method [37], using a MPO detection kit (Nanjing Jiancheng Bioengineering Institute). Colon tissues were washed in ice-cold physiological saline to remove fecal residues and weighed on analytical scale, then homogenized three times in 9 volumes of ice-cold physiological saline. The MPO activity was measured with a spectrophotometer (756pc Shanghai Spectrum Instrument Co. Ltd., China) by absorbance at 460 nm. MPO activity was defined as the quantity of enzyme degrading 1 µmol of peroxide per minute at 37°C and was expressed in units per gram weight of wet tissue.

### Immunohistochemical analysis

Colon tissues were fixed in 4% buffered paraformaldehyde and 4 µm sections were prepared from paraffin-embedded tissues. After deparaffinization in xylene and rehydration in a series of graded alcohol, endogenous peroxidase was quenched with 3.0% hydrogen peroxide in methanol for 30 min. For the heat rehabilitation of antigen, slides were immersed in 10 mmol/L citrate buffer (pH 6.0) and performed by microwave for 10 minutes, then cooled for 20 minutes. Slides were incubated with polyclonal primary antibody of Bax (diluted to 1∶100) overnight at 4°C. The sections were washed three times with phosphate-buffered saline (PBS) and incubated with polyclonal rabbit anti-mouse biotinylated secondary antibody (Dako, CA, USA) at room temperature for 30 min. After washing with PBS, the sections were incubated with 3, 3′-diaminobenzidine solution (Sigma, St Louis, MO, USA) until a brown reaction product could be visualized. Sections were washed with PBS and stained with haematoxylin. Then the sections were dehydrated with increasing concentration of ethanol, mounted with neutral gum, and observed under an Olympus BH-2 microscope.

### Analysis of intestinal epithelial cell apoptosis

Apoptotic epithelial cells in colonic tissue were analyzed using the terminal deoxynucleotidyl transferase (TdT)-mediated dUTP-biotin nick end labeling (TUNEL) assay according to the manufacturer's instruction. TUNEL-positive nuclei were clearly identified as brown-stained nuclei, which indicated the presence of DNA fragmentation due to apoptosis. TUNEL-positive cells were determined by observing 1000 cells in randomly selected fields.

### Western blot analysis

Colon tissues were pounded to pieces in liquid nitrogen, then disrupted by homogenization on ice in hypotonic lysis buffer containing: 20 mmol/L N-2-Hydroxyethylpiperazine-N′-2′-ethanesulfonic Acid (HEPES) pH 7.8, 1.5 mmol/L MgCl_2_, 420 mmol/L NaCl, 1 mmol/L ethylenediamine tetraacetic acid (EDTA), 1 mmol/L ethylene glycol bis (2-aminoethyl ether)-N,N,N′N′-tetraacetic acid (EGTA), 1 mmol/L dithiothreitol (DTT), 0.5 mmol/L phenylmethyl sulfonylfluoride (PMSF), 15 µg/ml trypsin inhibitor, 3 µg/ml pepstatin, 2 µg/ml leupeptin, 40 µmol/L benzidamin, 1% Nonidet P-40 and 20% glycerol. Homogenates were centrifuged at 4°C (12,000 g, 15 min). The supernatants were collected as nuclear extracts in aliquots and stored at −80°C for western blotting analysis. Protein concentrations were determined by Bradford assay.

Equal amounts of protein (40 µ*g*/lane) were separated on 10% sodium dodecylsulfate-polyacrylamide gel electrophoresis (SDS-PAGE) and transferred to a nitrocellulose membrane. Membranes were blocked in 5% skim milk dissolved in 10 mmol/L Tris-HCl, pH 7.5, 100 mmol/L NaCl and 0.1% Tween-20, and incubated with anti-NF-*κ*Bp65, anti-IκB*α*, anti-IKKβ, anti-β-actin (Santa Cruz Biotechnology) and anti-phosphserine IκB*α* polyclonal antibodies (Cell signaling, Beverly, USA) at dilution of 1∶500, 1∶500, 1∶500, 1∶1000 and 1∶500, respectively. The membranes were washed three times for 15 min with Tween-20/Tris-buffered saline (TTBS) and incubated with horseradish peroxidase-conjugated secondary antibodies (diluted to 1∶2000, Santa Cruz Biotechnology). Blots were again washed with TTBS, and then developed by enhanced chemiluminescence detection regents (ECL, Amersham). The protein bands were quantified by the average ratios of integral optic density (IOD) following normalization to the housekeeping gene.

### Real-time quantitative RT-PCR

Total RNA was isolated from colonic tissue using Trizol reagent (Invitrogen, Carlsbad, CA, USA). For each sample, 1 µg of RNA was reverse-transcribed (RT) using AMV reverse transcriptase (Promega), 1 mmol/L deoxyribonucleotide triphosphate (dNTP) (GibcoBRL), and oligo (dT_12–18_) 0.5 µg/µl (GibcoBRL). ABI TaqMan 2×PCR Master mix of primers (Applied Biosystems, Foster City, CA, USA) and TaqMan MGB probes (FAM dye-labeled) were used for the target genes and pre-developed 18S rRNA (VIC-dye-labeled probe). Real-time RT-PCR was performed using an ABI Prism 9700 sequence detection system (Applied Biosystems, Foster City, CA) with specific primers for rat NF-*κ*Bp65, IκB*α*, IKKβ, TNF-*α*, IL-8, TGF-β1, COX-2, Fas, FasL, Bcl-2, Caspase-3. β-actin was used as a house-keeping gene. All primers were designed by Primer Premier 5.0 software (Molecular Biology Insights, USA). The primer sequences used in PCR amplification are shown in Table 1. Thermal cycler parameters were as follows: one cycle of 50°C for 2 min, 95°C for 10 min, and 40 cycles of denaturation (95°C, 30 s) and combined annealing/extension (60°C, 30 s). Duplicate cycle threshold (CT) values were analyzed in Microsoft Excel using the comparative CT (^ΔΔ^C_T_) method as described by the manufacturer. The amount of target (2**^−ΔΔCT^**) was obtained by normalizing it to an endogenous reference (18S rRNA) and relative to a calibration curve.

**Table 1:** Sequences of the amplification primers used in the real-time RT-PCR.

mRNA Species		Oligonucleotides (5′→3′)
NF-*κ*Bp65	forward	ACCTGGAGCAAGCCATTAGC
	reverse	CGGACCGCATTCAAGTCATA
IκB*α*	forward	TGGAGCCGACCTCAATAAACC
	reverse	TGCGACTGTGAACCACGATG
IKKβ	forward	AGCTCTGGAACCTCCTGAAGA
	reverse	AGCTCCAGTCTAGGGTCGTGA
TNF-*α*	forward	GCCAATGGCATGGATCTCAAAG
	reverse	CAGAGCAATGACTCCAAAGT
IL-8	forward	CTCCAGCCACACTCCAACAGA
	reverse	CACCCTAACACAAAACACGAT
TGF-β1	forward	CGCAACAACGCAATCTATG
	reverse	CCCTGTATTCCGTCTCCTT
COX-2	forward	ACTACGCCGAGATTCCTGACA
	reverse	ACTGATGAGTGAAGTGCTGGG
Fas	forward	AACTTCTATTGCAATGCTTCTCTCTGT
	reverse	CAAGGCTCAAGGATGTCTTCAA
FasL	forward	CACCAACCACAGCCTTAGAGTATCA
	reverse	ACTCCAGAGATCAAAGCAGTTCCA
Bcl-2	forward	TCAAACAGAGGTCGCATGCT
	reverse	CATCTGCACACCTGGATCCA
Caspase-3	forward	GAGGCCGACTTCCTGTATGC
	reverse	TGACCCGTCCCTTGAATTTC
β-actin	forward	TGGAATCCTGTGGCATCCATGAAAC
	reverse	TAAAACGCAGCTCAGTAACAGTCCG

### Statistical analysis

The results were expressed as the mean ± SD. SPSS 16.0 statistical software was used for the analysis. Differences between groups were compared using one way analysis of variance and two-tailed Student's *t* test. *P<*0.05 was considered significant.

## Results

### Iridoid glycosides ameliorate DSS-induced colitis

**Figure 1:**
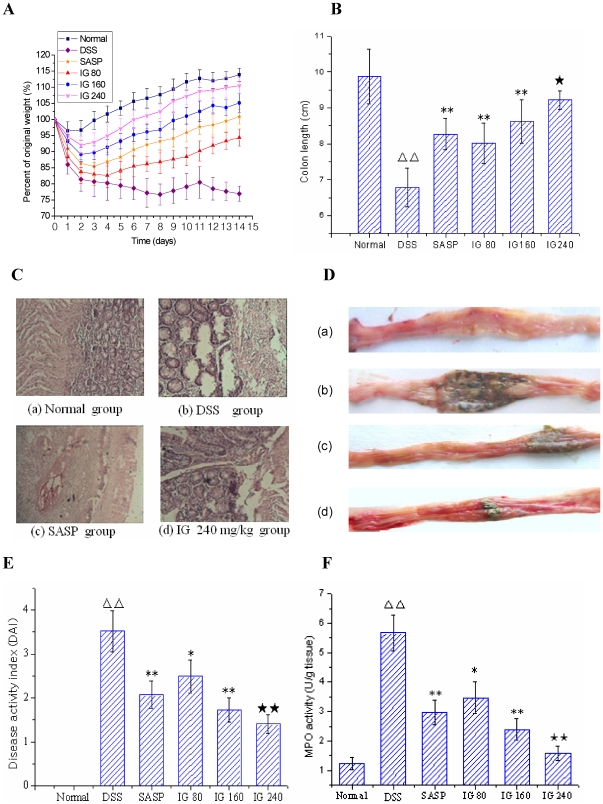
Iridoid glycosides (IG) ameliorate the clinical, macroscopic and histological features of acute colitis model. Colitis was induced by 4% DSS in drinking water. Rats were administrated p.o. at different dose of IG 80, 160 and 240 mg/kg twice daily for 14 days after administration of DSS. All rats were sacrificed on the 14 day after the first IG administration. The severity of colonic injury and the clinical evaluation were monitored by weight changes (A), colon length (cm) (B) histological analysis (C), macroscopic signs (D: a, normal group; b, DSS group; c, SASP group; d, high dose group of IG 240 mg/kg), disease activity index (E) and MPO activity (U/g tissue) (F). Representative macroscopic and histological analysis of rat colonic tissue in normal group, DSS group and high dose group of IG (240 mg/kg) (C and D) were shown in this figure. SASP (150 mg/kg p.o.) was used as a positive control. IG administration dose-dependently diminished these parameters. The data are expressed as the mean ± standard deviation (Mean ± SD). ΔΔ p<0.01 versus normal group; * p<0.05, ** p<0.01 versus DSS group; ★ p<0.05, ★★ p<0.01 versus SASP group.

The therapeutic efficacy of IG on experimental colitis was assessed by body weight change, colon length, disease activity index (DAI), histological analysis and MPO activity. Severe drug-induced colitis was observed from the 4th day after DSS administration, which was characterized by obvious hyperemia, edema, stool consistency and ulceration (Fig. 1C). The animals of normal group averagely increased (13.89±2.01)% in the body weight, whereas a dramatic body weight loss and shortened colon length accompanied with obvious diarrhea in DSS group was observed as a result of the colitis, and was maintained during the experimental period (Fig. 1A and B). Administration of SASP effectively suppressed body weight loss and colon shortening. However, treatment of IG (80–240 mg/kg) could significantly reverse the changes of two parameters induced by DSS in a dose-dependent manner. Histological studies showed that inflamed tissue had marked necrosis of colonic mucosa, hyperemia and adhesions to adjacent bowel wall. Additionally, MPO activity was significantly increased after DSS administration compared with normal group. Administration with SASP could improve the acute inflammatory response. Nevertheless, treatment with IG dose-dependently inhibited these pathological symptoms and MPO activity with lower DAI (Fig. 1C, D, E and F). In particular, IG (240 mg/kg) had a better therapeutic effect than SASP. This result strongly suggested that the inhibition of neutrophil infiltration was a mechanism for the protective effects of IG in experimental colitis.

### Effect of iridoid glycosides on key inflammatory cytokine expression

**Figure 2:**
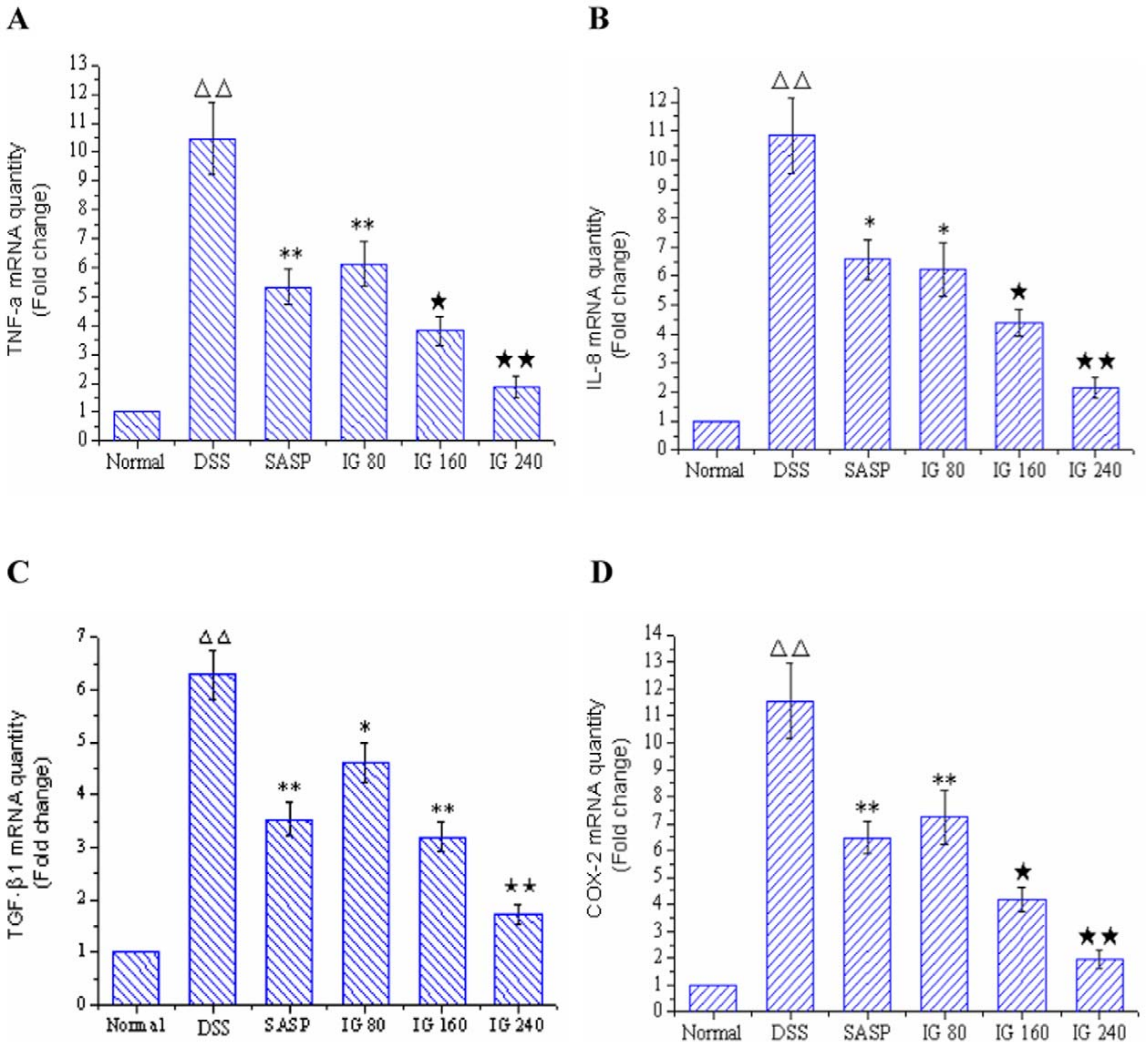
Effect of iridoid glycosides on the mRNA expressions of TNF-α, IL-8, TGF-β1 and COX-2 in DSS-induced colitis. Rats were administered with IG (80, 160, 240 mg/kg/day p.o.) for 14 days. SASP (150 mg/kg/day p.o.) was used as a positive control. The mRNA expressions of (A) TNF-α, (B) IL-8, (C) TGF-β1 and (D) COX-2 were determined using real-time quantitative PCR. The data are expressed as the mean ± standard deviation (Mean ± SD). ΔΔ p<0.01 vs normal group; * p<0.05, ** p<0.01 vs DSS group; ★ p<0.05, ★★ p<0.01 vs SASP group.

In order to test whether treatment with IG may modulate the inflammatory process through the regulation of the expressions of key NF-κB-dependent cytokines, the mRNA levels of TNF-α, IL-8, TGF-β1 and COX-2 in colonic tissue were examined by real-time quantitative RT-PCR. As shown in Fig. 2, the mRNA levels of TNF-α, IL-8, TGF-β1 and COX-2 in DSS model group showed a significantly high expression compared with normal control group (P<0.01). In contrast, administration of SASP obviously decreased the expressions of TNF-α, IL-8, TGF-β1 and COX-2 mRNA. Especially, IG treatment inhibited the expressions of the four inflammatory genes in a dose-dependent manner to some degree (Fig. 2 A, B, C and D). Maximum inhibition effect was observed with IG at a dose of 240 mg/kg.

### Effect of iridoid glycosides on IEC apoptosis

**Figure 3:**
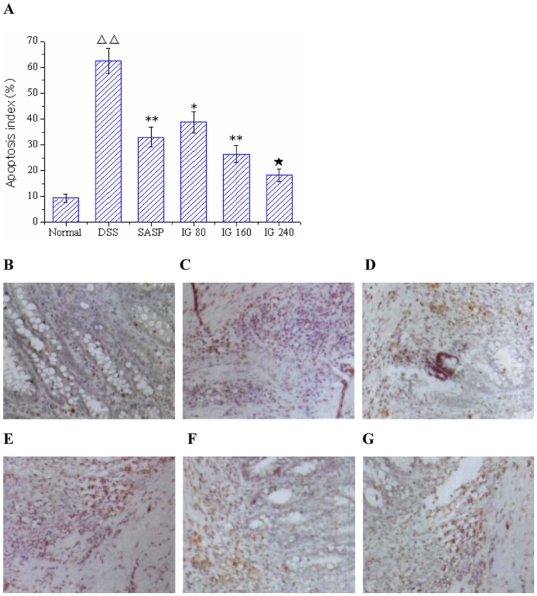
Effect of iridoid glycosides on intestinal epithelial cells apoptosis. Apoptotic cells in the colon tissues were detected using TUNEL assay. Cells with nuclei that stained dark brown were considered to be TUNEL-positive. (A) Apoptosis index (%); (B) Normal group, (C) DSS Model group; (D) SASP group; (E) IG 80 mg/kg, (F) IG 160 mg/kg and (G) IG 240 mg/kg (original magnification ×400). Apoptosis index indicated that the percentage of TUNEL-positive cells significantly decreased in a dose-dependent manner after treatment with IG compared with the DSS Model group. Data were expressed as mean ±SD (each group, n = 10). ΔΔ p<0.01 versus normal group; * p<0.05, ** p<0.01 versus DSS group; ★ p<0.05 versus SASP group.

To study whether experimental colitis is associated with IEC apoptosis and the effect of IG on IEC apoptosis, we measured TUNEL staining in colonic tissues. As shown in Fig. 3B, few apoptotic cells were observed in normal group, whereas colon tissues demonstrated a marked appearance of dark brown apoptotic cells and intercellular apoptotic fragments after treatment with DSS (P<0.01, Fig. 3C). In contrast, administration of SASP obviously decreased the number of apoptotic epithelial cells and apoptotic fragments (Fig. 3D). Treatment with IG remarkably reduced the percentages of TUNEL-positive cells in a dose-dependent manner (P<0.01, Fig. 3E, F and G). In particular, IG in a dose of 240 mg/kg was the most effective in suppressing IEC apoptosis.

### Effect of iridoid glycosides on the expressions of serial genes involved in IEC apoptosis

**Figure 4:**
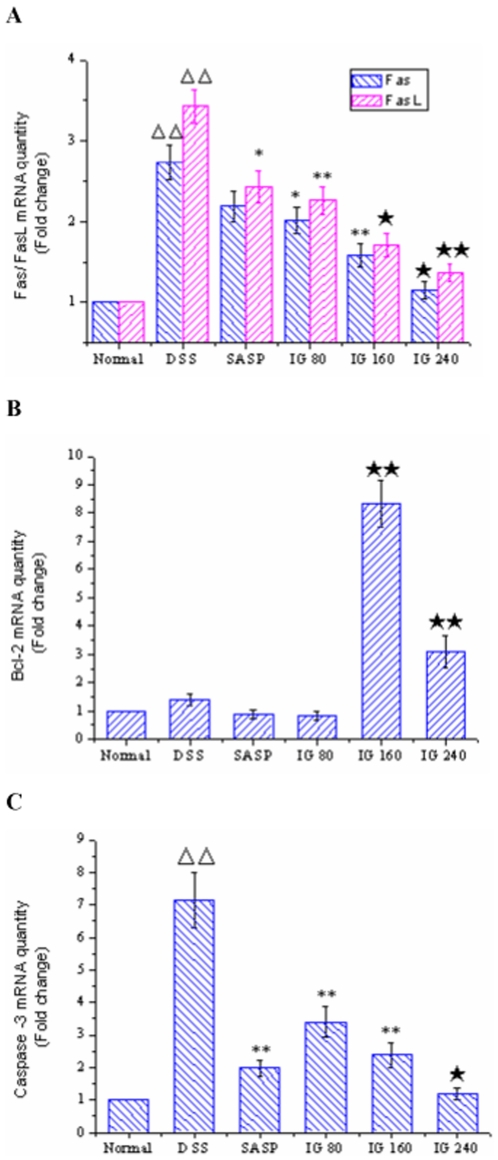
Effect of iridoid glycosides on the mRNA expressions of Fas/FasL, Bcl-2 and Caspase-3 in DSS-induced colitis. Rats were administered with IG (80, 160, 240 mg/kg/day p.o.) for 14 days. SASP (150 mg/kg/day p.o.) was used as a positive control. The mRNA expressions of (A) Fas/FasL, (B) Bcl-2 and (C) Caspase-3 were determined using real time quantitative PCR. The data are expressed as the mean ± standard deviation (Mean ± SD). ΔΔ p<0.01 vs normal group; * p<0.05, ** p<0.01 vs DSS group; ★ p<0.05, ★★ p<0.01 vs SASP group.

As it could be informed from Fig. 4A, the mRNA levels of Fas and FasL in normal group were very low. Both of their expression levels in the colonic epithelia of DSS-induced model group were significantly higher than those of normal group (p<0.01). In contrast, administration of SASP effectively inhibited FasL mRNA expression (p<0.05), whereas the mRNA level of Fas had no significant difference compared with DSS group (p>0.05). Treatment with IG dose-dependently decreased the mRNA expressions of Fas and FasL. Especially, IG in a dose of 240 mg/kg was the most effective in down-regulating the mRNA levels of Fas and FasL.

**Figure 5:**
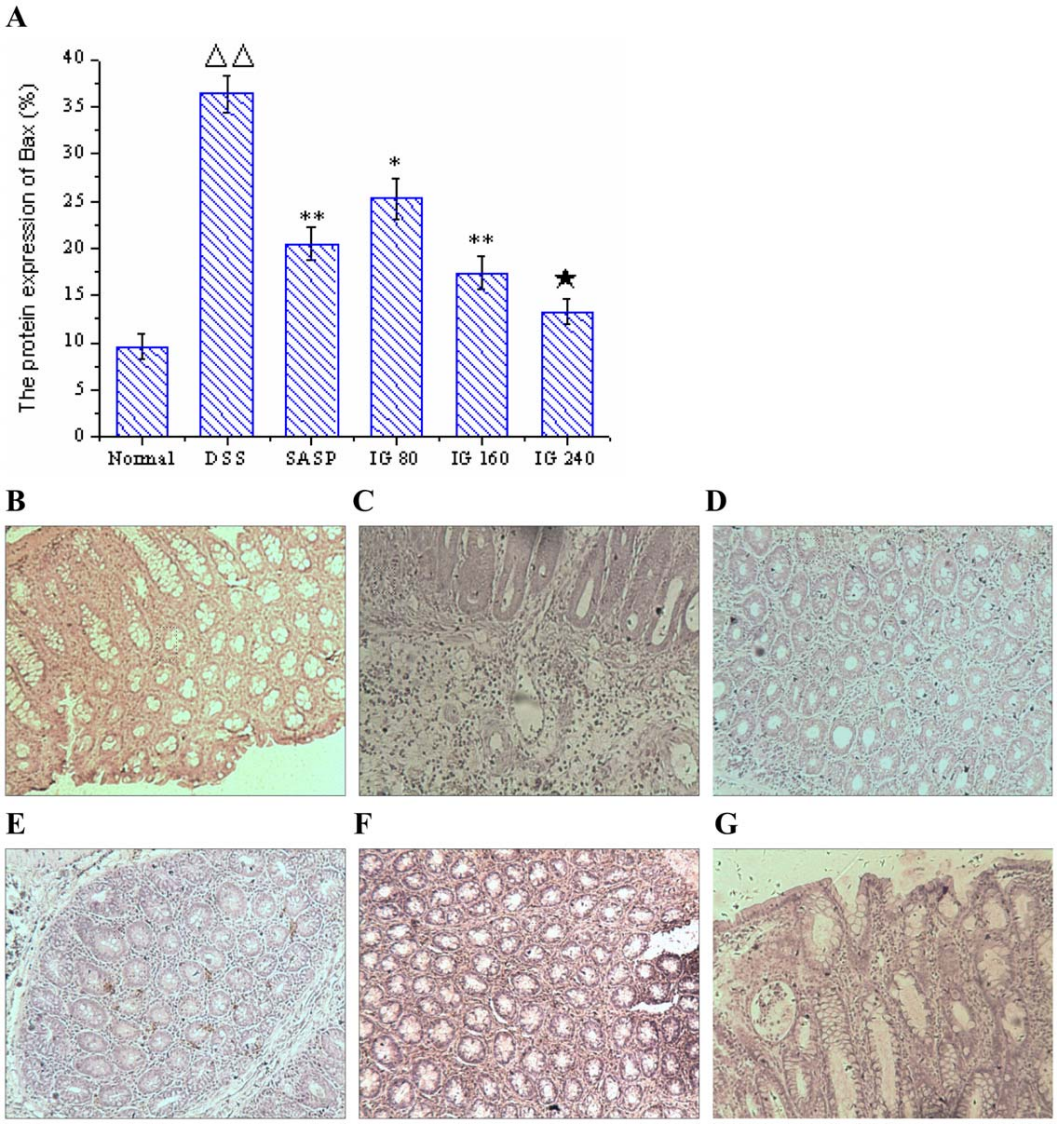
Immunohistochemical localization for Bax. There was few specific expression of Bax in normal tissue (B). Protein expression of Bax was significantly increased in the epithelial cell, intestine glands and in the inflammatory cells infiltrating in the tissue of model animals (C). Treatment of SASP (150 mg/kg), Bax protein expression was obviously reduced (D). Administration with IG (80 mg/kg, E; 160 mg/kg, F; 240 mg/kg, G), protein expression of Bax was effectively inhibited in both mucosa and submucosa in dose-dependent manner (A). Specific expression of Bax was very weak in the colonic tissue of high-dose group (**G**). Original magnification 400×.

Immunohistochemistry and real-time PCR results showed that the protein expression of Bax in DSS group was distinctly elevated compared with normal group (p<0.01, Fig. 5), whereas no significant difference of Bcl-2 mRNA level was found between DSS group and normal group (p>0.05, Fig. 4B). SASP obviously suppressed Bax protein expression level. But it had no effect on the mRNA expression of Bcl-2. Nevertheless, IG treatment significantly diminished the induced upregulation of Bax protein expressions in a dose-dependent manner. IG (240 mg/kg) was the most effective in reducing the protein expression of Bax (*P<*0.01, Fig. 5). In addition, IG could dose-independently up-regulate the mRNA level of Bcl-2 (*P<*0.01, Fig. 4B).

As shown in Fig. 4C, the Caspase-3 mRNA expression of colonic epithelia cells in DSS group was markedly increased compared with normal group (p<0.01). Administration of SASP effectively inhibited Caspase-3 mRNA expression (p<0.01). The Caspase-3 mRNA level of colonic epithelia in IG group was significantly decreased in a dose-dependent manner compared to that in DSS group. Furthermore, the effect of IG in a dose of 240 mg/kg was prior to that of SASP (p<0.05).

Our study suggested that one mechanism underlying the protective effect of IG involved in the regulation of serial genes associated with IEC apoptosis in experimental colitis.

### Effect of iridoid glycosides on NF-κB signal pathway

**Figure 6:**
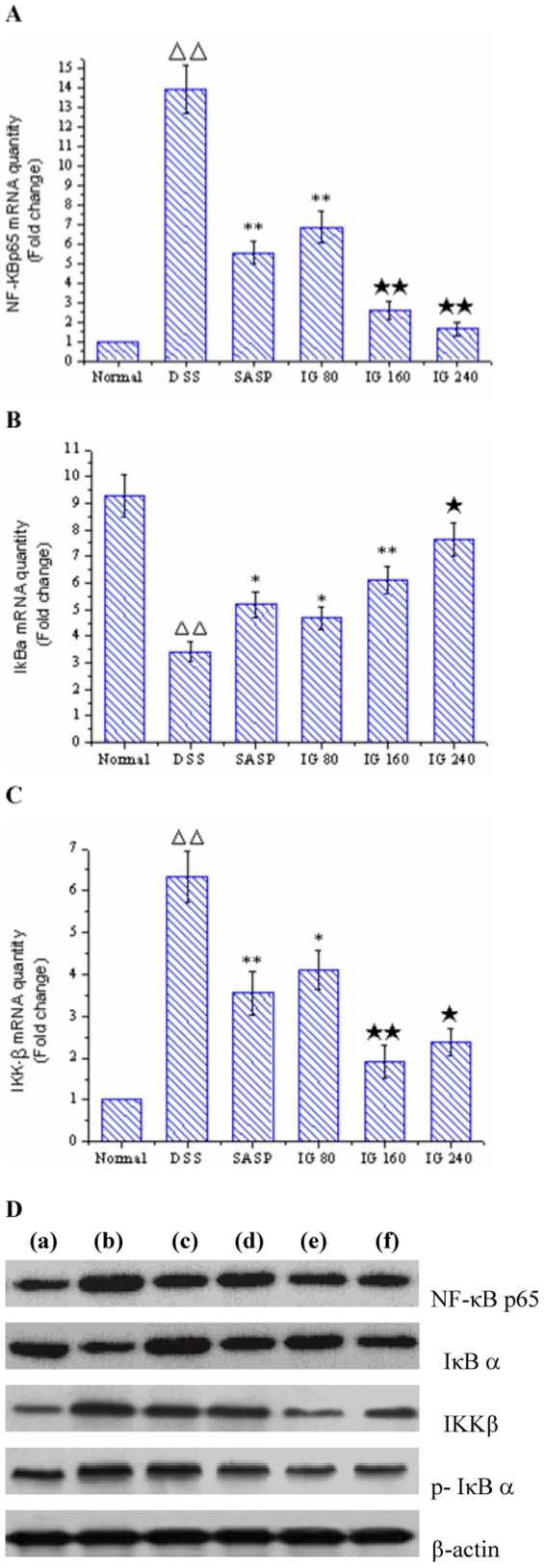
Iridoid glycosides blocks NF-κB activation by inhibiting NF-κB p65 mRNA expression, IκBα phosphorylation/degradation and IKKβ activity in rats with DSS-induced colitis. The mRNA levels of (A) NF-κB p65, (B) IκB α and (C) IKKβ in colonic tissues were determined using real time quantitative PCR. Total protein was extracted and examined for NF-κB p65, IκB α, phosphor-IκB α and IKKβ expression by Western blotting (D: a, normal group; b, DSS group; c, SASP group; d, low dose group of IG 80 mg/kg; e, middle dose group of IG 160 mg/kg; f, high dose group of IG 240 mg/kg). Densitometry was made to following normalization to the control (housekeeping gene). The results are representative of three experiments performed on different samples. Data are expressed as the mean ± standard deviation (Mean ± SD). ΔΔ p<0.01 vs normal group; * p<0.05, ** p<0.01 vs DSS group; ★ p<0.05, ★★ p<0.01 vs SASP group.

NF-κB activation can be regulated at several steps, including the nuclear translocation of its p65/RelA component and phosphorylation/degradation of IκBα [28]. In addition, IκBα phosphorylation is mediated by the activation of IKKβ unit of the IKK complex in numerous cell systems [23]. To further determine the impact of IG on cytokine-induced signal transduction in experimental colitis, we investigated the expression levels of representative upstream and downstream signal proteins involved in NF-κB activation using real-time PCR and Western blotting analysis. A significant increase in the mRNA and protein expressions of NF-κBp65 and IKKβ were observed in colonic epithelia cells of DSS-induced model group (p<0.01, Fig. 6A, C and D). Furthermore, the degradation and phosphorylation of IκBα were distinctively induced in colonic epithelia cells of DSS-induced rats compared with normal group (p<0.01, Fig. 6B and D). In contrast, administration of SASP obviously reduced NF-κBp65 and IKKβ expressions in DSS-induced colitis, and the phosphorylation/degradation of IκBα were effectively suppressed, too. In accordance with the data presented in Fig. 6A and D, IG inhibited NF-κB nuclear translocation as indicated by dose-dependently decreasing p65/RelA expression and IκBα degradation in colonic epithelia cells compared with that in DSS group. However, IG (240 mg/kg) exerted the optimum effect in suppressing NF-κBp65 level and IκBα degradation. On the other hand, IG significantly blocked IκBα phosphorylation and IKKβ activity in a dose-independent manner (p<0.05, Fig. 6C and D). IG (160 mg/kg) showed the maximum inhibition effect.

These results indicated that IG markedly blocked NF-κB signaling pathway in experimental colitis by inhibiting its binding to target DNA and suppressing IκBα phosphorylation/degradation and IKKβ activity in IEC.

## Discussion

The present study was undertaken to investigate the potential anti-inflammatory and anti-apoptosis effects of IG on experimental colitis induced by DSS and the mechanisms involved. NF-*κ*B signaling pathway regulates multiple κB-dependent genes involved in the inflammatory response and cell apoptosis and represents an ideal target for the molecular therapy of UC [23], [38]. Therefore, understanding the molecular mechanisms involved in this pathway is an essential step towards countering the damaging effects of pro-inflammatory mediators and cell apoptosis in IBD. In our previous study, IG, the main active fraction extracted from *F. syringae* leaves, has been known to possess strong anti-inflammatory activities and acts as an inhibitor for NF-κBp65 and oxidative-free radicals in experimental colitis [33]. However, the mechanism whereby IG inhibits NF-κB activity and IEC apoptosis has not been investigated. On the basis of the above, we hypothesized that IG might also modulate IκB/NF-κB pathway in DSS-induced colitis, through which it could inhibit intestinal inflammation and IEC apoptosis *in vivo*.

To confirm the validity of these hypotheses, first, we investigated the effect of IG on NF-κB-mediated inflammatory cytokine expression in DSS-induced colitis, which represents several characteristics resembling human UC. Recent studies have demonstrated increased production of pro-inflammatory cytokines including TNF-α, IL-1β, IL-6, IL-8, ICAM-1 and COX-2 in IBD that are known to play a key role in the modulation of intestinal immune system [29], [33]. TNF-α released from macrophages in the early inflammatory response plays an important role in experimental colitis and it is likely the regulator key of the inflammatory cascade in IBD. IL-6 and IL-8 could stimulate neutrophil chemotaxis and relate to the presence of necrosis in the colon which led to tissue destruction. COX-2 is an important NF-κB-dependent mediator, in both of acute murine colitis and colitis-related cancer. The COX-2 level was increased in IBD and in colon cancer [39], [40]. Therefore, blockade of these inflammatory mediators can offer an alternative therapy for UC. However, it is insufficient for achieving the optimal therapeutic effects only to block individual factors in a multifactorial inflammatory disease. Actually, individual factor such as cytokine or COX-2 only represents a downstream target, whereas NF-κB is just the final common pathway of the inducible expression of these pro-inflammatory genes or rate-limiting step in the inflammatory cascade of UC [33], [38]. Increased NF-κB activation has been detected in the intestinal lamina propria of patients with IBD, and in a acute murine colitis model [26], [29]. The cytokine-induced IκB/NF-κB signaling cascade is complex, involving the participation of multiple kinases and adapter proteins. The critical rate-limiting step in the activation of the NF-*κ*B pathway is the catalytic I*κ*B kinase (IKK) [41]. Cytokine (TNF-α and IL-1β) or bacterial product signaling converge on the IKK complex to trigger IκBα phosphorylation and ultimately NF-κB activity in numerous cell systems [4], [42]. Activation of NF-κB then upregulates the expression of numerous κB-dependent pro-inflammatory genes involved in intestinal inflammation, including TNF-α, IL-6, IL-8, ICAM-1, iNOS, and COX-2 [29], [43]. IKK is made up of two kinases, IKKα and IKKβ. Whereas IKKα is activated by an only limited set of stimuli, IKKβ activation occurs upon receptor-mediated stimulation by a broad set of microbial or host-derived ligands [44]. Gene depletion studies have demonstrated that IKKβ, but not IKKα, plays an essential role in NF-κB activation [41]. In order to achieve the optimal therapeutic effects, the application of a therapeutic strategy that interferes with NF-*κ*B pathway (the upstream target) in the cascade of inflammation, namely, the blockade of simultaneously the expression of multiple pro-inflammatory genes, might be more effective than suppressing individual factor in treatment of UC. To demonstrate the effect of IG on NF-κB pathway, we utilized real-time quantitative PCR and Western blotting to determine NF-κBp65 level, IKKβ activity and IκBα phosphorylation/degradation in colonic tissue. The results indicated that the protein and mRNA expressions of NF-*κ*Bp65 and IKKβ were significantly increased in DSS-treated rats. NF-*κ*Bp65 mRNA expression was dose-dependently inhibited and IKKβ decreased dose-independently after administration of IG for 2 weeks. Moreover, DSS strongly induced IκBα phosphorylation and triggered IκBα degradation in colonic tissue. IG also blocked IκBα phosphorylation/degradation in a dose-dependent manner. On the other hand, our study indicated that the levels of TNF-*α*, IL-8 and COX-2 in DSS group were increased more distinctly than normal group and reduced dose-dependently after treatment with IG in response to NF-*κ*Bp65 and IKKβ activity. In addition, the effect of IG at dose of 160 mg/kg and 240 mg/kg were all prior to SASP. Since the promoter regions of TNF-*α*, IL-8 and COX-2 had also been shown to contain consensus binding motifs for NF-*κ*B. In the present study, we firstly infer that anti-inflammatory effect of IG may be linked with inhibition of multiple pro-inflammatory genes through blockade of IκB/NF-κB pathway in experimental colitis.

Second, to clarify whether IG could modulate IEC apoptosis in UC *in vivo*, we investigated the effect of IG on the relative expression level of a series of apoptosis genes in DSS-induced colitis. Recent evidence suggests that NF-*κ*B activates the transcription of many genes capable of regulating apoptosis, known as the “cell-death substrates” [45]. Fas/FasL is an important pathway of involved in the induction of epithelial cell apoptosis in UC [38]. When UC occurred, the expression of FasL is markedly upregulated on the surface of infiltrating cytotoxic lymphocytes in active UC and binds the Fas receptor on the basolateral epithelial membrane [46]. Fas associated death domain recruits molecules, including procaspase-8, to form the death-inducing signaling complex [47]. Procaspase-8 is then cleaved into its activated form, and the apoptotic cascade ensues, culminating in the activation of executioner caspase-3 [48]. These interactions accelerate migration and activity of neutrophils, induce generation of excessive ROS through NF-κB activation and expression of FasL, and inhibit immune response at the inflammatory site, resulting in progressive mucosal lesion of UC [38], [46]. Anti-apoptotic NF-*κ*B target genes include TGF-β1, inhibitors of apoptosis proteins (IAP), and prosurvial members of the Bcl-2 gene kindred and so on [19], [21]. As Bcl-2 could prolong the life of cells, it has been generally accepted as an anti-apoptosis gene. The apoptosis promoting gene Bax is a new member of Bcl-2 gene kindred, it could form a dimer with Bcl-2 to inhibit its function [49]. The relative expression ratio of Bax/Bcl-2 determines whether apoptosis occurs in IEC or not. Excessive expression of Bax promotes apoptosis, and when the expression of Bcl-2 gained advantage, the cells would continue to exist [50]. Among the anti-apoptotic regulatory proteins, TGF-β1 plays especially important role in the pathogenesis of IBD [51]. TGF-β1 expression can be induced by pro-inflammatory cytokines, such as IL-1β and TNF-α [21]. The general opinion showed that TGF-β1 expression in plasma is increased parallel to the increase in cytokine secretion due to inflammation in patients with UC and Crohn's disease (CD), which can be used as a marker for differential diagnosis of the active phase of both diseases [52]. Additionally, plasma TGF-β1 levels are affected by anti-inflammatory drugs such as glucocorticoid, sulfasalazine and 5-aminosalicylic acid [52], [53]. The present study confirmed that DSS-induced colitis leads to a substantial increase of IEC apoptosis. Meanwhile, the mRNA and protein expressions of Fas/FasL, Bax and caspase-3 in colonic epithelia were significantly increased. Activation of these apoptosis genes increased the number of apoptotic cells, which might be one of the important mechanisms of colonic pathological changes in UC. After treatment with IG, activation of Fas/FasL, Bax and caspase-3 in colonic epithelia were markedly downregulated compared with DSS group, and the number of apoptosis cells was also decreased. In addition, our results showed persistent inflammation resulted in a significant upregulation of TGF-β1 expression and reduction of Bcl-2 expression. IG treatment dose-dependently reduced TGF-β1 level and increased the mRNA and protein expression of Bcl-2. These findings demonstrate, for the first time, the novel protective effects of IG against inflammation in colon, suggesting a potential clinical value in the treatment of IBD. Considering that the common effector pathway for regulating expression of these pro-inflammatory cytokines and apoptosis genes is IκB/NF-κB transduction system, IG could ameliorate experimental colitis through the blockade of NF-κB signaling.

Although IG had anti-inflammatory functions through acting on the IκB/NF-κB pathway, and these effects most probably contributed to its therapy of UC. Many upstream signaling proteins and downstream cytokines of NF-κB in which treatment of IG were also involved have not been completely demonstrated. The pro-inflammatory gene transcriptional program mediated by IκB/NF-κB pathway was induced by toll-like receptor (TLR) activation and subsequent recruitment of MyD88, followed by p38-dependent transcriptional activity of the NF-κB complex leading to induction of target genes, such as TNF-a, IL-27, IL-15, MMP-9, and VCAM-1 [43], [54]. Therefore, our future studies will investigate these upstream targets and other downstream cytokines, in order to elucidate underlying molecular mechanisms responsible for the therapeutic effects of IG. Despite we demonstrated the protective effects of IG on preventing acute colitis in DSS-induced murine model, which has proven useful for examining the underlying pathophysiology of IBD. It should be further validate that whether IG would modulate the inflammation in different colitis models such as the IL-10 knock-out model and therapeutic trails for established colitis [43]. In addition, NF-κB activation has emerged as a hallmark for many human hematologic malignancies and solid tumors, most commonly because of persistent activation of the IKK complex [55], and IKK could be related to the development of colitis-associated cancer [56]. Intriguingly, a recent study demonstrated that 5-aminosalicylic acids (5-ASAs) are the most commonly prescribed anti-inflammatory drugs for IBD. Regular 5-ASA intake may reduce the risk of colorectal cancer in patients with IBD through anti-NF-κB action by direct inhibition of IKK [57]. In our study, IG ameliorates experimental colitis through inhibiting NF-κB pathway by blockade of IKK activity. In view of this, the effect of IG on colitis-associated cancer should be further clarified by *in vitro* and *in vivo* studies to support this hypothesis.

In conclusion, the present results demonstrated that IG dose-dependently ameliorated the severity of DSS-induced colitis. The possible mechanisms in the protective effect of UC were concluded that IG could inhibit multiple pro-inflammatory molecules and modulate serial genes involved in IEC apoptosis through blocking NF-κB signal pathway. Additionally, numerous studies from clinical trial as well as animal studies have shown that few toxic effects or side effects are found with treatment of IG [58]. Recently, antisense oligonucleotide against NF-*κ*B has been applied in the treatment of UC. However, its price was expensive and the safety need to be further identified. Therefore, IG, as natural inhibitor of NF-*κ*B signaling pathway, could be a promising remedy for the treatment of IBD.
